# Effect of systemic high dose enzyme replacement therapy on the improvement of CNS defects in a mouse model of mucopolysaccharidosis type II

**DOI:** 10.1186/s13023-015-0356-0

**Published:** 2015-10-31

**Authors:** Sung Yoon Cho, Jeehun Lee, Ah-Ra Ko, Min Jung Kwak, Sujin Kim, Young Bae Sohn, Sung Won Park, Dong-Kyu Jin

**Affiliations:** Department of Pediatrics, Samsung Medical Center, Sungkyunkwan University School of Medicine, 81 Irwon-ro, Gangnam-gu, Seoul, 135-710 Republic of Korea; Clinical Research Center, Samsung Biomedical Research Institute, Seoul, Republic of Korea; Department of Pediatrics, Pusan National University Hospital, Pusan National University School of Medicine, Busan, Republic of Korea; Department of Pediatrics, Myongji Hospital, Seonam Univeristy College of Medicine, Goyang, Republic of Korea; Department of Medical Genetics, Ajou University Hospital, Ajou University School of Medicine, Suwon, Republic of Korea; Department of Pediatrics, Dankook University College of Medicine, Cheil General Hospital & Woman’s Health Care Center, Seoul, Republic of Korea

**Keywords:** Mucopolysaccharidosis type II, Hunter syndrome, Central nervous system, Enzyme replacement therapy, Iduronate-2-sulfatase, Hunterase®

## Abstract

**Background:**

Mucopolysaccharidosis type II (MPS II, Hunter syndrome), is caused by a deficiency of iduronate-2-sulfatase (IDS). Despite the therapeutic effect of intravenous enzyme replacement therapy (ERT), the central nervous system (CNS) defects persist because the enzyme cannot cross the blood-brain barrier (BBB). There have been several trials of direct infusion to the cerebrospinal space showing promising results; however, this approach may have limitations in clinical situations such as CNS infection. The objective of this study was to improve the CNS defect with systemic high-dose ERT.

**Methods:**

Systemic ERT was performed using three doses (1, 5, and 10 mg/kg weekly) of IDS for three different durations (1, 3, and 6 months) in IDS knock out (KO) mice of two age groups (2 months, 8 months). GAG measurement in tissues, brain pathology, and behavioral assessment were analyzed.

**Results:**

Brain IDS activities increased in parallel with the concentrations of IDS injected. The glycosaminoglycan (GAG) level and histopathology in the brains of the young mice improved in a dose- and duration-dependent manner; however, those were not improved in the old mice, even at higher doses of IDS. The spontaneous alternation behavior was recovered in young KO mice treated with ≥ 5 mg/kg IDS; however, no significant improvement was observed in old KO mice.

**Conclusions:**

These results suggest that high-dose ERT given to mice of earlier ages may play a role in preventing GAG accumulation and preventing CNS damage in IDS KO mice. Therefore, ERT above the present standard dose, starting in early childhood, could be a promising treatment regimen for reducing neurological impairment in Hunter syndrome.

**Electronic supplementary material:**

The online version of this article (doi:10.1186/s13023-015-0356-0) contains supplementary material, which is available to authorized users.

## Background

Mucopolysaccharidosis (MPS) type II, also known as Hunter syndrome [OMIM 309900], is a rare, X-linked lysosomal storage disorder. The estimated incidence of MPS is 1 per 162,000 live births [1], and MPS II is the most common type of MPS, accounting for 54.6 % of documented cases in Korea [2]. The disease is caused by a deficiency of iduronate-2-sulfatase (IDS) (EC 3.1.6.13), which is involved in lysosomal glycosaminoglycan (GAG) catabolism. Patients with MPS II show a broad spectrum of symptoms, including dysmorphic facial features, hepatosplenomegaly, skeletal deformities, joint stiffness, severe retinal degeneration, and hearing impairment. Progressive deterioration of the central nervous system (CNS) is observed in the severe phenotype of MPS II.

The therapeutic effects of current enzyme replacement therapy (ERT) for MPS II include improvements in joint mobility, gait, and pulmonary and respiratory functions, and reductions in liver and spleen volumes and urinary GAG excretion [3–7]. However, current ERT using the labeled dose (0.5 mg/kg weekly) does not yield any neurocognitive improvement because it cannot pass the blood-brain barrier (BBB). CNS degenerative changes in MPS II are devastating and must be resolved to improve the quality of life in patients with severe forms of Hunter syndrome. Thus, it is imperative to develop efficient therapies that treat the CNS symptoms of Hunter syndrome.

In our previous study, a pseudotyped recombinant adeno-associated virus type 2/8 vector encoding the human IDS gene was administered intravenously to IDS knockout (KO) mice. This experiment involved liver-specific gene therapy and indicated the effectiveness of systemic high-dose therapy because the high level of enzyme expression in the liver induced a high level of enzymes in the blood [8].

Few studies using high-dose ERT have been conducted using the KO mouse model of Hunter syndrome with different study designs [9, 10]. Polito et al. [9] demonstrated that systemic ERT allowed IDS to reach the brain and, thus, to correct substantially the CNS pathology of MPS II mice and to improve their neurobehavioral features. However, GAG levels in the brain were not analyzed in their study. Our group previously suggested that early, high-dose ERT attenuated ventriculomegaly and histological abnormalities in the brains of IDS KO mice. However, neurobehavioral assessment or IDS activity analysis was not performed, and mice at a single age (4 weeks) were only included in that study [10].

In the present study, systemic high-dose ERT was investigated in an IDS KO mouse model at different starting ages and ERT doses and durations. This study demonstrates that the systemic administration of human IDS to young IDS KO mice enables the enzyme to decrease the GAG accumulated in the brain, correct many neurodegeneration markers, and alleviate the neurobehavioral CNS disease phenotype.

## Methods

### Animals

All animal experiments were performed with the approval of the Institutional Animal Care and Use Committee at the Laboratory Animal Research Center, Samsung Biomedical Research Institute (Seoul, Korea). A previously reported IDS KO mouse model [8] was used for the MPS II animal model. Briefly, the KO mice were prepared by replacing exons 2 and 3 of the IDS gene with the neomycin resistance gene. Carrier females were bred with male mice with a B6/129 background strain to produce heterogeneous females, hemizygous male KO mice, wild-type (WT) males, and female littermates. The genotypes of all mice were confirmed via polymerase chain reaction using DNA obtained from a tail snip. The C57BL6 strain was used for the WT control mice. All animals in this study were male.

### Idursulfase beta

For ERT, recombinant human IDS beta (Hunterase®, Green Cross Corp., Yongin, Korea) was used and administered to the IDS-ERT group by intravenous injection. The idursulfase beta treatment is well tolerated in Korean patients with MPS II and results in significantly reduced urinary GAG excretion and improved 6-min walk test results in 0.5 mg/kg and 1 mg/kg weekly treatment groups [11]. Hunterase® was concentrated by centrifugation in Amicon Ultra Centrifugal Filters (Millipore, Bedford, MA, USA) to a final concentration of 4.75 mg/mL. The preparations were diluted in 100 μL phosphate-buffered saline. Different doses of Hunterase® were injected weekly over a 10–15-s period into the lateral tail vein of the mice, and an equal volume of normal saline was injected in an identical manner into the WT and KO control groups.

### Study design

The study protocol is shown in Table 1. Because the mouse BBB is known to be maturated at 6 weeks old [12], we used 8-week-old mice in the young mouse group.

**Table 1:** Study protocol

Group (start age)	Subgroup	Dose (mg/kg)	Weekly infusion	Duration (mo)	Assessment	Age (mo)
A (2 mo)	*n* = 7	10	4 times	1	Tissue GAGs	3
					Brain pathology	3
	*n* = 7	5				
	*n* = 7	1				
					Footprint analysis	3
	*n* = 7	1 + mannitol				
KO	*n* = 5	Saline				
WT	*n* = 5	Saline				
B (2 mo)	*n* = 7	10	24 times	6	Tissue GAGs	8
					Brain pathology	8
	*n* = 7	5				
	*n* = 7	5 + mannitol			Footprint analysis	8
	*n* = 7	10 (2mo) → 5 (4mo)				
	*n* = 7	10 (2mo) → 2 (4mo)			Y-maze analysis	8
KO	*n* = 5	Saline				
WT	*n* = 5	Saline				
C (8 mo)	*n* = 7	10	12 times	3	Tissue GAGs	11
	*n* = 7	5			Brain pathology	11
	*n* = 7	1			Footprint analysis	11
KO	*n* = 5	Saline			Y-maze analysis	11
WT	*n* = 5	Saline				

*KO* knockout, *WT* wild type, *mo* month(s)

Group A (short-term-treated young mouse group): A group of young IDS KO mice (aged 2 months; *n* = 28; seven mice per group) received Hunterase® at doses of 10, 5, or 1 mg/kg, or 1 mg/kg with 25 % mannitol (0.1 ml) pretreatment via the tail vein weekly for 1 month. Because mannitol has been shown to facilitate the entry of peripherally delivered therapeutic substances to the CNS by disrupt the BBB transiently [13], the mannitol pretreatment group was added.

Group B (long-term-treated young mouse group): A group of young IDS KO mice (aged 2 months; *n* = 35; seven mice per group) received Hunterase® at doses of 10 or 5 mg/kg or 5 mg/kg with 25 % mannitol (0.1 ml) pretreatment via the tail vein weekly for a total period of 6 months. In addition, two dose-change subgroups were added (10 mg/kg for the first 2 months, then 5 or 2 mg/kg for the subsequent 4 months).

Group C (mid-term treated old mouse group): A group of old IDS KO mice (aged 8 months; *n* = 21; seven mice per group) received Hunterase® at doses of 1, 5, or 10 mg/kg (0.1 ml) via the tail vein weekly for 3 months.

Age-matched IDS KO (*n* = 5) and WT (*n* = 5) mice were used as controls for each group. The WT and IDS KO control groups were injected weekly with normal saline for the same period. After treatment, the animals were sacrificed via an intraperitoneal injection of Zoletil (50 mg/kg) and xylazine (10 mg/kg). Transcardiac perfusion was performed with ice-cold 0.9 % saline, and harvested tissues were stored at −80 °C until biochemical analysis was conducted.

### Preparation of tissue extracts for the analysis of GAG levels and quantitative analysis of GAG accumulation

Tissue extracts were prepared by homogenizing tissues in phosphate-buffered saline (PBS) using a tissue homogenizer. After hemisection in the midsagittal plane of brain, the half of whole brain was assessed for GAG levels and another half of whole brain was assessed for pathology. Homogenates were centrifuged at 20 000 × g for 30 min, and supernatants were collected. The total protein concentration (mg/ml) was assayed with a bicinchoninic acid (BCA) assay (Pierce, Rockford, IL, USA). GAG concentrations in tissue homogenates and urine were quantified using a colorimetric assay (Kamiya Biomedical Co., USA) according to the manufacturer’s instructions, and absorbance was measured at 620 nm using a chondroitin 6-sulfate standard curve. The GAG levels in tissue extracts were adjusted based on the total protein concentration, which was measured with the BCA assay, and are expressed as μg GAG per mg protein. GAG levels in urine were normalized to the creatinine content.

### IDS enzyme activity assay

The homogenates from the brains were obtained after perfusion of saline. The IDS assay was performed as previously described [14]. IDS activity was measured in homogenates from the brains of treated mice (10 weeks of age) at 4 h after a single injection with doses of 1, 5, or 10 mg/kg (each dose, *n* = 3). To perform a valid comparison of the specific enzyme activity, three individual batches were analyzed. The enzyme activity was measured by determining the rate of enzyme-catalyzed hydrolysis of a synthetic substrate, 4-methylumbelliferyl-α-L-iduronide-2-sulfate-Na2 (MU-αIdoA-2S), in 50 mM of sodium acetate and 500 μg/mL bovine serum albumin at pH 5.5. The total protein concentration was measured with a BCA assay kit (Pierce, Rockford, IL, USA). The released 4-methylumbelliferyl (4MU) was quantified by measuring the fluorescence (excitation 355 nm, emission 460 nm, Victor X4, PerkinElmer, Waltham, MA) against a 4MU standard curve.

### Histological analyses

After the perfusion of the mice with ice-cold normal saline, their tissues were collected and fixed with 4 % paraformaldehyde overnight at 4 °C. The next day, the tissues were embedded in paraffin (Sigma0Aldrich, St Louis, MO, USA) after dehydration through a 70–100 % ethanol gradient. Finally, the paraffin blocks were cut to a thickness of 4 μm. Lysosomal-associated membrane protein-2 (Lamp-2) immunoreactivity is a lysosomal protein marker used for the detection of lysosomal storage disorders. For the immunohistochemical detection of Lamp-2, sections were treated with 10 % normal goat serum (Dako, Carpinteria, CA) for 20 min at room temperature to block nonspecific binding. Subsequently, sections were incubated with rat anti-Lamp-2 monoclonal antibody (1/100; Santa Cruz Biotechnology, Santa Cruz, CA) for 30 min at room temperature. After washing in PBS, the sections were incubated for 30 min at room temperature with HRP-labeled, polymer-conjugated secondary antibodies against rat IgG (Lamp-2). A color reaction was developed using ready-to-use 3,30-diaminobenzidine substrate-chromogen solution (Dako, Carpinteria, CA) for 5 min followed by washing with distilled water. Finally, the nuclei were lightly counter-stained with Mayer’s hematoxylin for 1 min. The hematoxylin-eosin staining of sections was performed according to standard protocols. Images of each section were captured with a magnifier digital camera using a Nikon ECLIPS 80i FL Upright Microscope (Nikon, Melville, NY) and were saved as JPEG files.

### Footprint pattern analysis

Footprint pattern analysis was performed as previously described in all groups to assess motor coordination [15, 16]. The mice were assessed at 3, 8 and 11 months in group A, B and C, respectively, before being sacrificed after their last injection. Gait abnormalities were assessed by painting the paws of the mice with non-toxic, washable paint and then placing the mice in a corridor lined with white paper. The forepaws were dipped in red ink, and the hindpaws were dipped in blue ink. All mice were trained for 3 days to walk across a white sheet of paper without stopping. The mice were assessed before being sacrificed after their last injection. Both the treated and control mice were tested during the same sessions to minimize variation.

### Spontaneous alternation behavior assessed by the Y-maze test

As another behavioral test, a Y-maze test was performed to assess spatial working memory in groups B and C. The Y-maze test has been reported to be useful for measuring short-term memory, general locomotor activity, and stereotypical behavior patterns [17–20]. The spontaneous alternation behavior Y-maze test uses a horizontal maze (30 cm long and 5 cm wide, with 12-cm-high walls) with three arms (labeled A, B, and C). The maze floor and walls are constructed of dark gray, polyvinyl plastic. Mice are initially placed within one arm, and the number of alternations (i.e., consecutive entry sequences of ABC, CAB, or BCA but not BAB) and the number of arm entries were manually recorded for each mouse over a 5-min period. The percentage of alternations was calculated according to the following equation: Percentage alternation = [(Number of alternations)/(Total arm entries-2)] × 100. The number of arm entries per trial was used as an indicator of locomotor activity.

### Statistical analysis

All results are expressed as the mean ± standard deviation. Statistical significance was determined for the compared measurements via one-way analysis of variance (ANOVA) tests, followed by Bonferroni’s correction. *P*-values <0.05 were considered significant. Stata software (ver. 11.0, Stata Corp LP, College Station, TX, USA) was used for all analyses.

## Results

During ERT, all animals in this study were alive and displayed no significant changes in behavior or vital signs. There were no significant differences in body weight or food consumption between the groups during the study, and no treatment-related adverse events were identified.

### Footprint pattern analysis

**Fig. 1:**
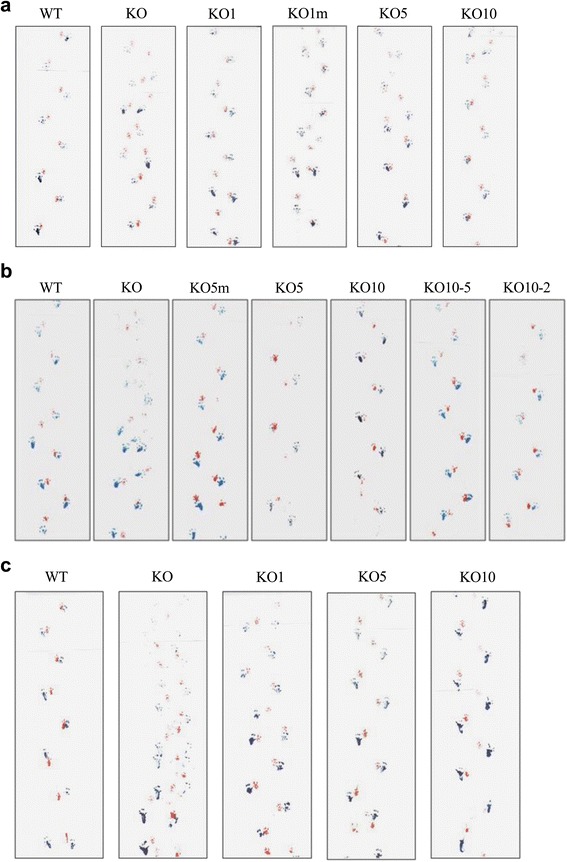
The walking footprint patterns of all of the mice at the end of the experiment. The walking footprint patterns show the forepaws (*red*) and hindpaws (*blue*) of each mouse of groups A (**a**), B (**b**), and C (**c**). Most WT mice walked in a straight line away from the investigator. By contrast, the IDS KO mice had an impaired walking pattern; their gait was slow, and they tended to walk in an unstable manner, with a short-stepped gait of approximately half the stride length of WT mice. The KO mice treated with any dose of IDS showed a trend towards amelioration of these abnormalities in all groups

The walking footprint patterns of all mice at the end of the experiment are illustrated (Fig. 1a, b and c). Most WT mice walked in a straight line away from the investigator. By contrast, the IDS KO mice had an impaired walking pattern. Their gait was slow, and they tended to walk in an unstable manner with a short-stepped gait characterized by strides of approximately half the length of those of WT mice; furthermore, they did not walk in a straight line in the tunnel, and their anterior and posterior paw prints did not overlap. The KO mice treated with any dose showed a trend towards amelioration of these abnormalities in all groups [A (Fig. 1a), B (Fig. 1b), and C (Fig. 1c)]. Each treatment group, even the KO mice treated with 1 mg/kg in groups A and C, showed an improved walking pattern; upon closer inspection, the mice treated with 5 mg/kg or 10 mg/kg showed slightly longer strides than the KO mice treated with 1 mg/kg in group C.

### Spontaneous alternation behavior assessed by the Y-maze test

**Fig. 2:**
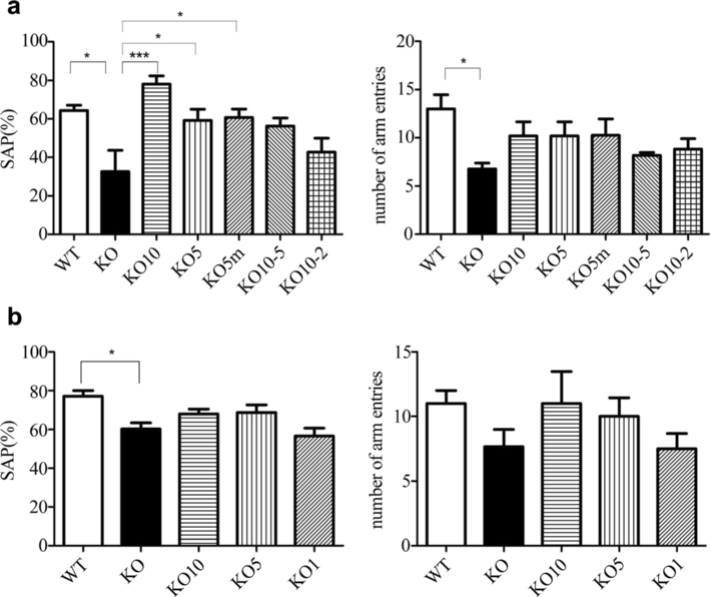
Effects of ERT on the spontaneous alternation behavior Y-maze test in groups B and C. In (**a**) and (**b**), the left picture shows the percentage of spontaneous alternations, and the right picture shows the number of entries. In group B, the KO group significantly decreased their spontaneous alternation behavior compared with the WT group (**a**, *p* < 0.05). This behavior was recovered in the KO mice treated with 10 mg/kg, 5 mg/kg, or 5 mg/kg plus mannitol pretreatment (*p* < 0.05) without significantly changing the number of arm entries. The number of arm entries by KO mice was significantly decreased compared with that by the WT mice (*p* < 0.05). The two dose-change groups showed a trend towards improved spontaneous alternation behavior; however, these changes were not statistically significant. In group C, the KO group significantly decreased their spontaneous alternation behavior compared with the WT group (**b**, *p* < 0.05). No treated mice of group C showed any significant improvement of spontaneous alternation behavior

The spontaneous alternation behavior percentage (SAP) of groups B and C are presented in Fig. 1d and e. In group B, the KO group had significantly decreased SAP compared with the WT group (Fig. 2a, *p* < 0.05). This behavior was recovered in the KO mice treated with 10 mg/kg, 5 mg/kg, or 5 mg/kg plus mannitol pretreatment (*p* < 0.05) without significantly changing the number of arm entries. The number of arm entries for KO mice was significantly decreased compared with that of the WT mice (*p* < 0.05). The two dose-change groups showed a trend of improving spontaneous alternation behavior; however, these changes were not statistically significant. In group C, the KO group displayed significantly decreased SAP compared with the WT group (Fig. 2b, *p* < 0.05). None of the treated mice in group C showed a significant improvement of SAP.

### GAG accumulation in brain tissue

**Fig. 3:**
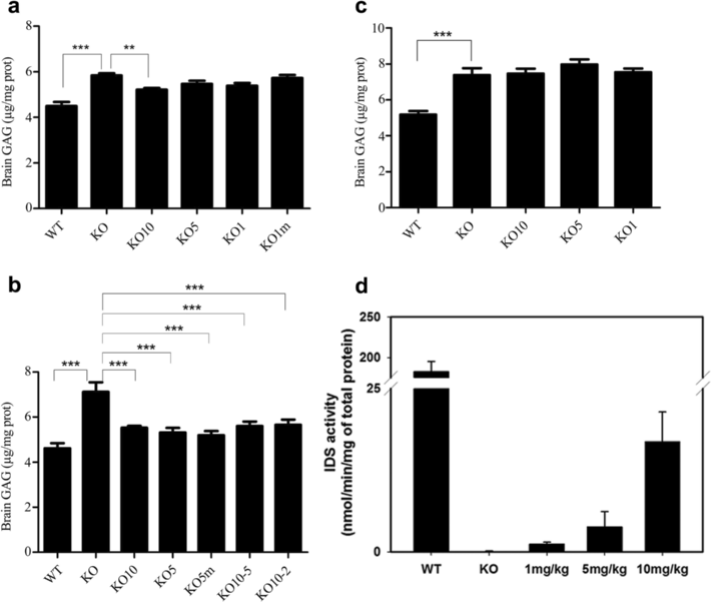
Measurement of total GAG and IDS activity in the brain. In all groups, the brain tissues of untreated KO mice showed significantly increased GAG concentrations compared with those of WT mice (*p* < 0.05). In group A, the IDS KO mice treated with 10 mg/kg IDS had significantly reduced brain GAG levels compared with the untreated IDS KO mice (*p* < 0.05) (**a**). In group B, compared with the untreated KO mice group, all treated groups showed significantly decreased brain GAG concentrations (*p* < 0.05) (**b**). In group C, no significant changes in brain GAG levels were found in any of the treated groups at any dose when treatment was started later (at 8 months) (**c**). IDS activity was measured in homogenates from the brains of treated mice (10 weeks of age) at 4 h after a single injection with doses of 1, 5, or 10 mg/kg (each dose, *n* = 3) (**d**). Brain IDS activities increased in parallel with the concentrations of IDS injected in the IDS KO mice. The data are the mean ± standard deviations. *p* < 0.05 versus untreated IDS KO control (ANOVA test). (***p < 0.001, **p < 0.01)

The measured GAG concentrations in the brain tissues of each group are presented in Fig. 3. In group A, the brain tissues of the untreated KO mice group showed significantly increased GAG concentrations compared with those of the WT mice (*p* < 0.05). No significant difference in brain GAG levels was observed between the IDS KO mice and the IDS KO mice treated with a dose lower than 10 ng/kg. However, the IDS KO mice treated with 10 mg/kg had significantly reduced brain GAG levels compared with the IDS KO mice (*p* < 0.05) (Fig. 3a). In group B, the brain tissues of the untreated KO mice group showed significantly increased GAG concentrations compared with the WT mice (*p* < 0.05). All treated groups with different doses showed significantly decreased brain GAG concentrations (*p* < 0.05) compared with the untreated KO mice (Fig. 3b). Even the 5 mg/kg group and the dose-change groups showed some reduction of brain GAG levels when ERT was started at a young age (2 months), after a 6-month treatment period. In group C, the mid-term-treated old mouse group, the brain tissues of the untreated KO mice group showed significantly increased GAG concentrations compared with the WT mice (*p* < 0.05). No significant change of brain GAG levels was found in any of the treated groups at any dose (Fig. 3c). IDS replacement therapy, even with as high of a dose as 10 mg/kg, did not reduce brain GAG levels if treatment was initiated at an old age (8 months).

### GAG accumulation in the other tissues and in urine

Accumulated GAG was markedly cleared from visceral tissues such as the liver and kidney of all treated mice in all groups. Accumulated GAG was markedly cleared in the urine of all treated mice in group A (Additional file 1: Figure S1).

### IDS enzyme activity assay

The brain IDS activity was extremely high in the WT mice, and brain IDS activities increased in parallel with the concentrations of IDS injected in the KO mice (Fig. 3d) (*p* < 0.05). Even in the IDS KO mice treated with the lowest IDS dose (1 mg/kg), the IDS activities measured in the brains were higher than those of the untreated IDS KO mice.

### Immunohistochemical analysis of brain tissue with Lamp-2

**Fig. 4:**
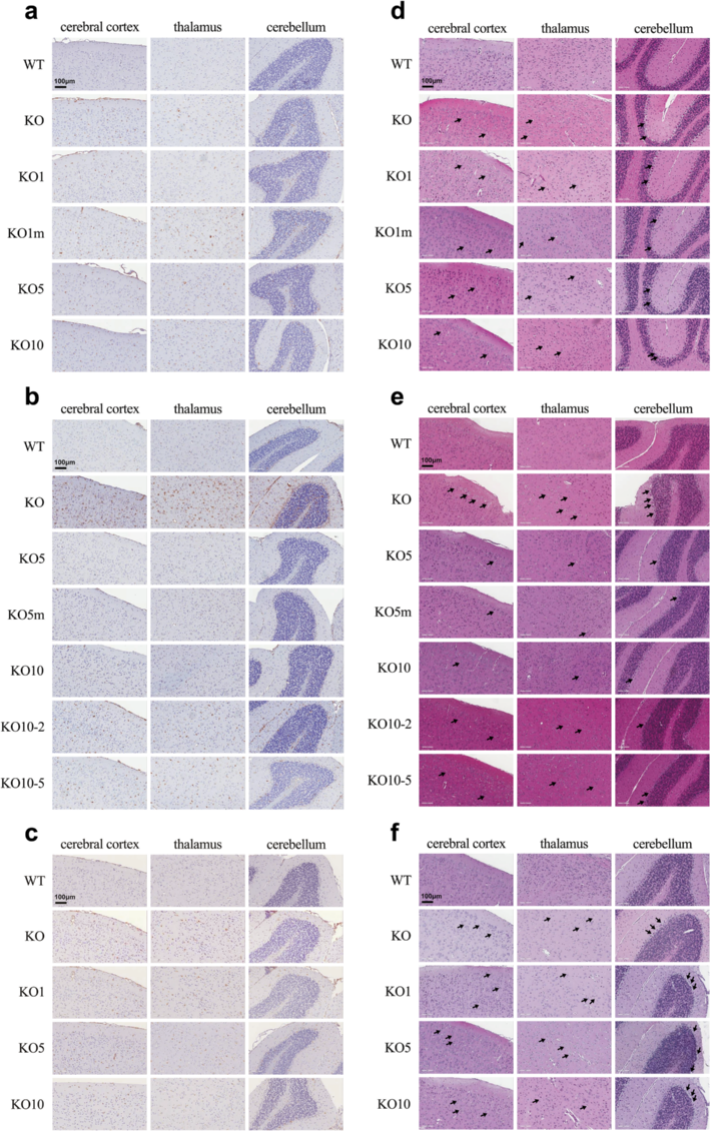
Histology of brain tissues such as the cerebral cortex, thalamus, and cerebellum with anti-Lamp-2 and H&E (200X). In group A, the untreated KO mice showed increased anti-Lamp-2 immunostaining compared with the WT mice. No treatment group showed reduced anti-Lamp-2 immunostaining (**a**). In group B, Lamp-2 immunostaining was evident in all areas of the untreated KO mice. A definite reduction in Lamp-2 immunostaining was observed in all treated groups at 5 mg/kg, 5 mg/kg plus mannitol pretreatment, or 10 mg/kg. The two dose-change groups did not reveal remarkable change in Lamp-2 immunostaining (**b**). In group C, the area of Lamp-2 immunostaining was less massive in the untreated MPS II mice compared with that of the untreated KO mice of group B. There was no significant reduction of Lamp-2 immunostaining in any treatment group (**c**). The number of distinct and enhanced neuronal vacuolation (arrow) in perivascular cells and neurons was markedly increased in the untreated KO groups of groups A, B, and C. Dyslamination and decreased cellular density were noted in the cortical area of the untreated KO groups. In group A, the reduced vacuolation was not remarkable in all treatment groups, even in the KO mice treated with 10 mg/kg after 4 weeks of ERT (**d**). In group B, the degree of vacuolation was more severe than in the untreated KO mice of group A. After 24 weeks of high-dose ERT (5 mg/kg/week or higher), the brain tissues of the KO mice showed a reduced degree of vacuolation, which was not remarkable in the two dose-change groups (**e**). In group C, the brain tissues of the treated KO mice did not show reduced vacuolation after 12 weeks of ERT (**f**)

Figure 4a, b and c displays the representative brain histological features of the cerebral cortex, thalamus, and cerebellum stained with Lamp-2 for each group. In group A, the untreated KO mice showed increased anti-Lamp-2 immunostaining, which marks lysosomal storage, compared with the WT mice. No treatment group, even the IDS KO mice treated with a 10 mg/kg dose, showed reduced anti-Lamp-2 immunostaining (Fig. 4a). In group B, Lamp-2 immunostaining was evident in all areas of the untreated KO mice. Lamp-2 deposition was more remarkable in the untreated KO mice of group B (8 months) than that of group A (3 months). A definite reduction in Lamp-2 immunostaining was observed in all groups treated with 5 mg/kg, with 5 mg/kg plus mannitol pretreatment, and with 10 mg/kg. The two dose-change groups did not reveal any change in Lamp-2 immunostaining (Fig. 4b). In group C, the area of Lamp-2 immunostaining was smaller in the untreated MPS II mice compared with that of the untreated KO mice of group B. This can be explained by decreased Lamp-2 deposits due to the decreased neuronal density in the older KO mice of group C. There was no significant reduction of Lamp-2 immunostaining in any treatment group (Fig. 4c).

### Hematoxylin and eosin staining of brain tissue

Figure 4d, e and f displays representative brain histology of the cerebral cortex, thalamus, and cerebellum stained with hematoxylin and eosin for each group. In group A, after 4 weeks of ERT, the reduction of vacuolation in the cerebral cortex, thalamus, and cerebellar cortex was not remarkable for all treatment group, even in the KO mice treated with 10 mg/kg IDS (Fig. 4d). In group B, the untreated KO mice (8 months) had substantial widespread cellular storage vacuoles presenting in the perivascular cells and neurons in the cerebral cortex, thalamus, and cerebellar cortex, which were more severe than in the untreated KO mice of group A (3 months). In group B, after 24 weeks of high-dose ERT (≥5 mg/kg weekly), the brain tissue of the KO mice showed reduced vacuolation in the cerebral cortex, thalamus, and cerebellar cortex (Fig. 4e), which was not remarkable in the two dose-change groups. In group C, after 12 weeks of ERT, the brain tissues of the treated KO mice did not show a reduction of vacuolation in the cerebral cortex, thalamus, or cerebellar cortex (Fig. 4f).

## Discussion

This study was designed to explore systemic ERT regimens and identify the optimal dosage, time of ERT initiation, and treatment period to protect the brain from degeneration using IDS KO mice. Results show that the early initiation and maintenance of systemic high-dose ERT can ameliorate brain tissue damage and prevent the progression of CNS defects.

In 2-month-old IDS KO mice, pathologic brain lesions were clearly observed. A systemically infused weekly high-dose (10 mg/kg) of IDS for 1 month was effective at resolving the accumulation of brain GAG; however, it was not markedly effective at improving brain histopathology. This could be explained by 1 month being too short a period to resolve brain histopathology. Using a high-dose IDS for 6 months (≥5 mg/kg weekly) brought about not only decrease of brain GAG accumulation but also amelioration of pathology of cerebral cortex, thalamus and cerebellar cortex. In contrast to the high-dose-treated group (5 and 10 mg/kg/week), the dose-transition group (10 to 5 and 10 to 2 mg/kg/week) showed less prominent amelioration of brain histopathology. This finding suggests that high-dose ERT for a certain time (over 4 months) is required to maintain the improvement of brain pathology. The mannitol pretreatment for disrupting BBB did not show any remarkable difference. Mannitol has been known to desrupt BBB transiently, however, it might not be enough for large molecule such as IDS enzyme to pass the BBB.

In 8-month-old mice, high-dose ERT did not decrease the brain GAG levels nor ameliorate brain histopathology. However, accumulated GAG was completely cleared from visceral tissues such as the liver and kidney in all treated mice. The initiation of treatment in old mice did not improve the CNS pathology because the neurodegeneration was too advanced. Polito et al. described that even a relatively low dose of IDS with both short and prolonged treatment times ameliorated the CNS defects in young IDS KO mice and that a high-dose of IDS ameliorated the CNS defects in old IDS KO mice [9]. Therefore, our observations are opposed to those of Polito et al. in terms of the age that ERT should be started and the dose/duration of ERT required for neuroprotection. Above all, our study analyzed brain GAG levels as the end product as well as brain pathology to provide comprehensive evidences of the action of the enzyme passing through the BBB.

In this study, the brain IDS activities in IDS-treated KO mice were higher than those of the untreated IDS KO mice; however, the IDS activities in IDS-treated KO mice were much lower compared with those measured in the WT mice. These results correspond with those of a previous study [9] and suggest that a small amount of enzyme in brain can improve the CNS pathology of IDS KO mice.

Systemic high-dose ERT also showed improvement in behavioral tests evaluating motor coordination and short-term memory. The IDS KO mice had an impaired walking pattern, which can be caused by limitation of joint motion or poor coordination. The KO mice treated with any dose of IDS showed a trend towards amelioration of these abnormalities in all groups. These results can be explained by both the improvement of joint motion and the decrease of accumulated GAG in the brain. The improvement of walking pattern in old IDS KO mice may rather be due to systemic effects of ERT on the muscles and joints as well as improved coordination.

Spontaneous alternation behavior was analyzed with the Y-maze test for groups B and C. The untreated KO mice showed significantly decreased alternation behavior, as previously reported [16]. However, in the present study, the number of arm entries was decreased in the untreated KO mice compared with the WT mice, in contrast to the previous report [16]. This finding suggests that the KO mice may have poor physical activity due to characteristics such as joint contracture and their tendency to hesitate. Spontaneous alternation behavior was recovered in young KO mice treated with 5 mg/kg or higher IDS; however, no significant improvement was observed in old KO mice, even those treated with 10 mg/kg of IDS.

At present, how IDS crosses the BBB to reach the brain remains unknown. There has been increasing evidence that when high serum levels of lysosomal enzymes are achieved through either gene therapy or ERT, a small fraction of the enzyme is able to cross the BBB [21–23]. First, one possible mechanism that may contribute to the transcytosis of these water–soluble proteins is pinocytosis [24]. Second, injection-dependent hydrodynamic effects due to rapid enzyme infusion in IDS KO mice cannot be excluded. However, this is less likely because only ~100 μL of volume was administered by tail-vein injection in the present study. Third, the extracellular pathway that has been shown to allow small quantities of molecules as large as albumin to cross the BBB represents a possible mechanism [25]. Finally, it is possible that residual mannose-6- phosphate receptor or other uncharacterized receptors more efficiently facilitate the transport of IDS in the context of high plasma enzyme levels. Further studies addressing the mechanism of crossing the BBB will perhaps be important for modifying the IDS enzyme to promote increased uptake into the brain. We also suggest that a next generation form of IDS with enhanced activity should be developed to improve CNS defects.

There was no administration-related hypersensitivity responses with high-dose ERT in the MPS II mice. For applying to humans, there are several possible adverse effects to be considered, including the immediate immune reaction such as hypersensitivity reactions or production of circulating antibodies. Preparing for the hypersensitivity reaction and monitoring the antibodies levels will assure optimal treatment. In addition, the cost of high-dose ERT will be very high; therefore, determining the optimal dose and interval should be considered to minimize the cost in a following study. In order to provide early treatment to patients with Hunter syndrome, early diagnosis is most important. Newborn screening of Hunter syndrome, therefore, should be considered.

In the present study, the low IDS level in brain could decrease of brain GAG level, which suggests high-dose intermittent treatment could be possible. This study is meaningful in terms of the potential clinical application of these findings to improve CNS problems of Hunter syndrome. Although intraventricular [16] and intrathecal [26, 27] enzyme or gene vector administration [8, 28] have produced neurological improvements, these strategies are limited by their invasive nature. Intrathecal enzyme administration to patients is particularly difficult due to poor patient cooperation or to device dysfunction. Intraventricular administration can bypass the BBB; however, the systemic effects of this technique remain in question, and it requires specialized neurosurgical skills. In one Japanese study [29], hematopoietic stem cell transplantation (HSCT) in patients with Hunter syndrome showed effectiveness towards brain involvement, when performed before signs of brain atrophy appear. However, the disadvantages of HSCT are the mortality and morbidity associated with the transplantation procedure and difficulty of finding of suitable donors.

Several systemic high-dose ERT researches have been conducted in other lysosomal disease animal models. Most studies [9, 23, 30–36] showed reduced neuropathology, however, one study [37] did not. Overall, the inefficiency of the CNS delivery in normal-dose ERT may be circumvented by high-dose ERT.

## Conclusions

In conclusion, the early application of high-dose ERT may play a role in preventing GAG accumulation, thus reducing ensuing brain damage in MPS II mice. Therefore, it is suggested that ERT above the current standard dose started as early as possible could be a promising treatment regimen for reducing neurological impairment by preventing brain GAG accumulation in patients with severe Hunter syndrome. The mannitol pretreatment did not show any remarkable difference. Further study to identify the ideal IDS dose and protocol to sufficiently treat the CNS phenotype of MPSII mice is required.

## Additional file

Additional file 1: Figure S1. Measurement of total GAG in other tissues and in urine. (DOCX 607 kb)

## Abbreviations

MPS: Mucopolysaccharidosis
IDS: Of iduronate-2-sulfatase
GAG: Glycosaminoglycan
CNS: Central nervous system
ERT: Enzyme replacement therapy
BBB: Blood-brain barrier
KO: Knockout
WT: Wild-type
PBS: Phosphate-buffered saline
BCA: Bicinchoninic acid
Lamp-2: Lysosomal-associated membrane protein-2
SAP: Spontaneous alternation behavior percentage
