# Supplementary material for: Effect of systemic high dose enzyme replacement therapy on the improvement of CNS defects in a mouse model of mucopolysaccharidosis type II
Source: Orphanet J Rare Dis. 2015 Oct 31;10:141. doi: 10.1186/s13023-015-0356-0 (PMC4628320; doi:10.1186/s13023-015-0356-0)

**Additional file 1: Figure S1 Measurement of total GAG in other tissues and in urine.**

Accumulated GAG was markedly cleared from the livers of all of the treated mice in groups A (Supp Fig. S1A), B (Supp Fig. S1B), and C (Supp Fig. S1C). Accumulated GAG was markedly cleared from the kidneys of all of the treated mice in groups A (Supp Fig. S1D), B (Supp Fig. S1E), and C (Supp Fig. S1F). Accumulated GAG was completely cleared from the urine of all of the treated mice in group A (Supp Fig. S1G).

Supp Fig. S1A


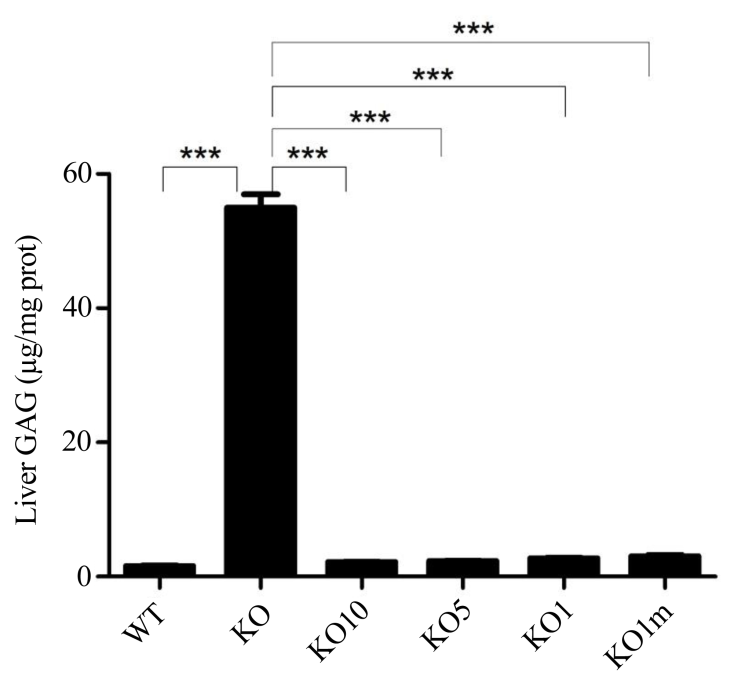


Supp Fig. S1B


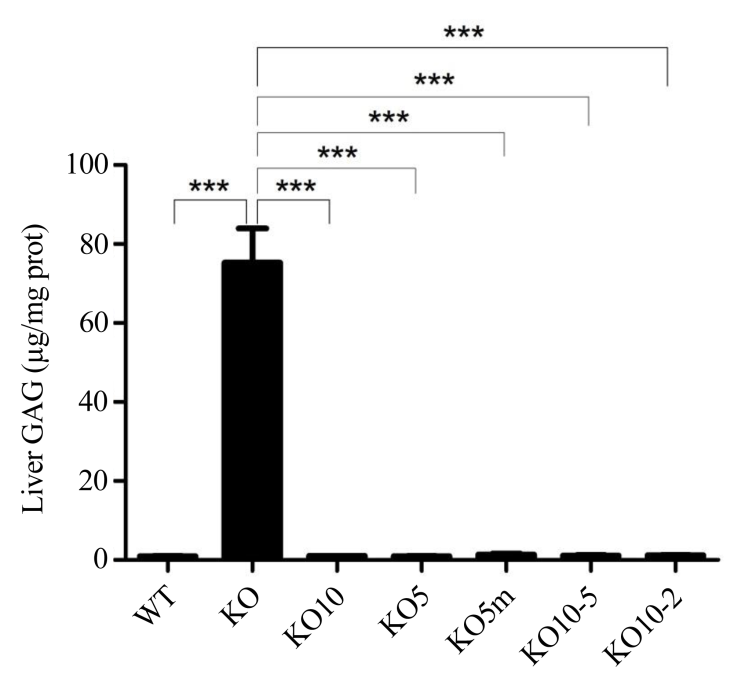


Supp Fig. S1C


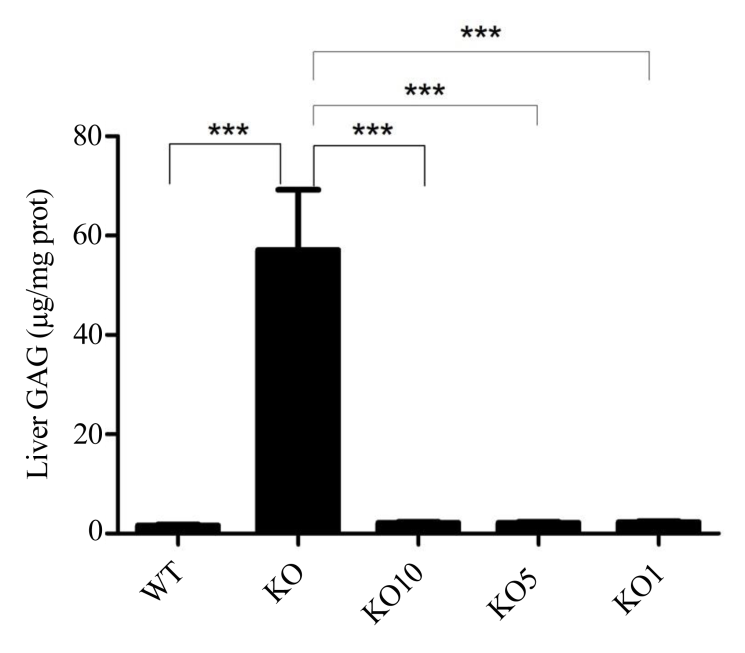


Supp Fig. S1D


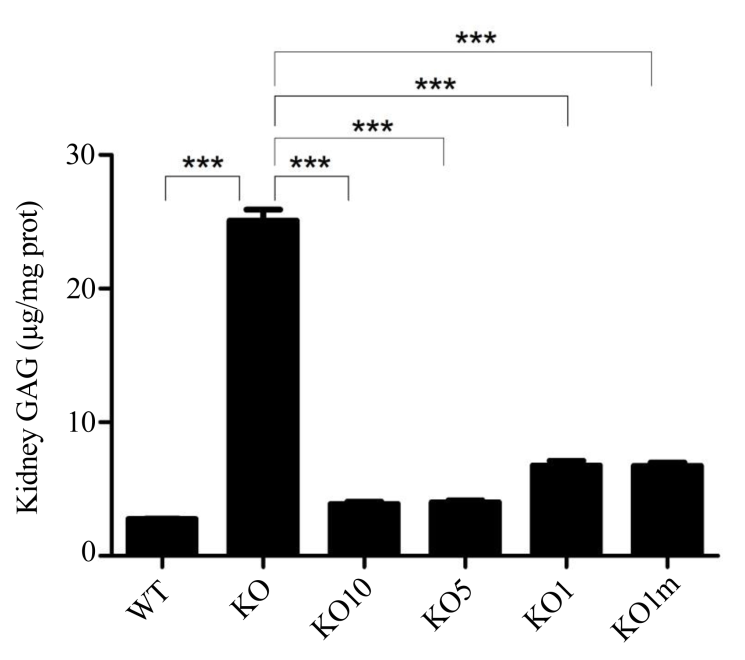


Supp Fig. S1E


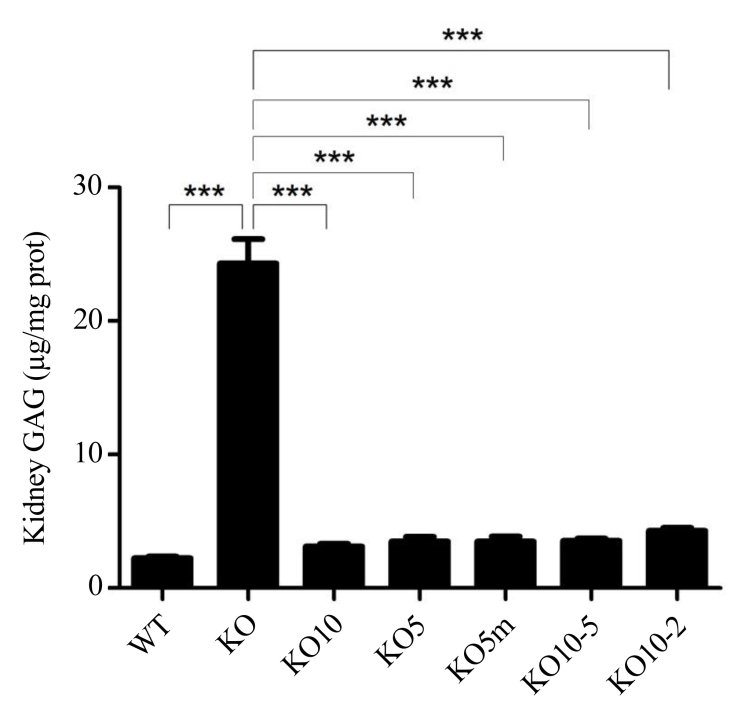


Supp Fig. S1F


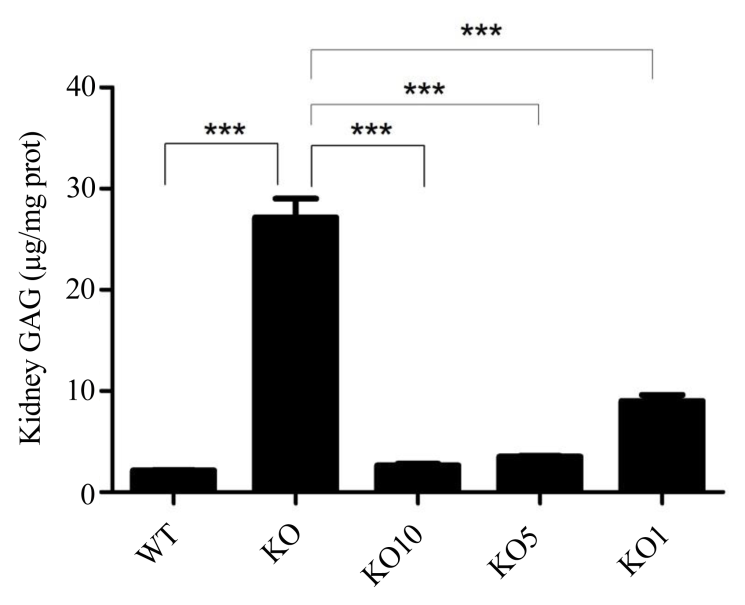


Supp Fig. S1G


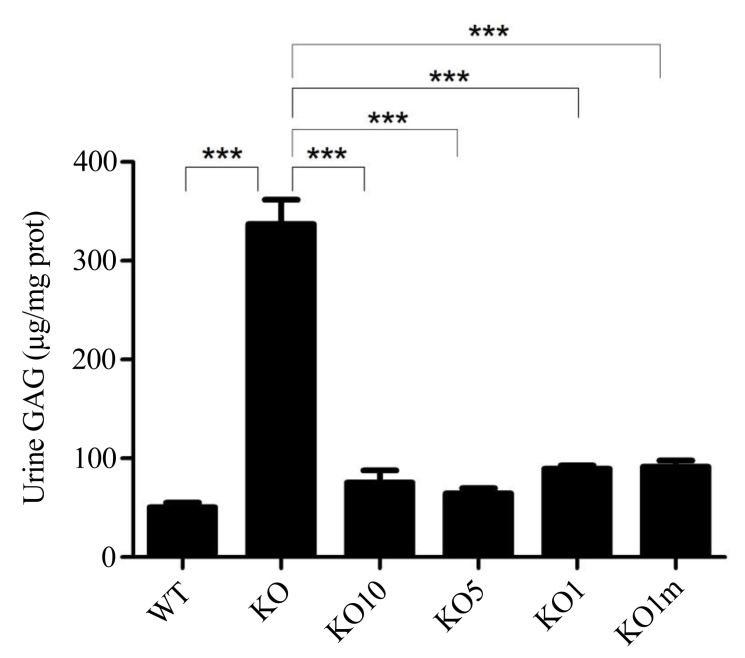

Supplement: Additional file 1: Figure S1. — Measurement of total GAG in other tissues and in urine. (DOCX 607 kb) [file 13023_2015_356_MOESM1_ESM.docx]
